# Higher PD-L1 Immunohistochemical Detection Signal in Frozen Compared to Matched Paraffin-Embedded Formalin-Fixed Tissues

**DOI:** 10.3390/antib10030024

**Published:** 2021-06-22

**Authors:** Hazem Ghebeh, Fatmah A. Mansour, Dilek Colak, Akram A. Alfuraydi, Amal A. Al-Thubiti, Dorota Monies, Monther Al-Alwan, Taher Al-Tweigeri, Asma Tulbah

**Affiliations:** 1 Stem Cell & Tissue Re-Engineering Program, King Faisal Specialist Hospital and Research Centre, Riyadh 11211, Saudi Arabia; fattu.mans@gmail.com (F.A.M.); aalfuraydi@ksu.edu.sa (A.A.A.); aalthubiti@kacst.edu.sa (A.A.A.-T.); MAlwan@kfshrc.edu.sa (M.A.-A.); 2 College of Medicine, Al-Faisal University, Riyadh 11533, Saudi Arabia; 3 Department of Biostatistics, Epidemiology and Scientific Computing, King Faisal Specialist Hospital and Research Centre, Riyadh 11211, Saudi Arabia; DColakkaya@kfshrc.edu.sa; 4 Department of Genetics, King Faisal Specialist Hospital and Research Centre, Riyadh 11211, Saudi Arabia; DMonies@kfshrc.edu.sa; 5 Oncology Centre, King Faisal Specialist Hospital and Research Centre, Riyadh 11211, Saudi Arabia; ttwegieri@Kfshrc.edu.sa; 6 Department of Laboratory Medicine and Pathology, King Faisal Specialist Hospital and Research Centre, Riyadh 11211, Saudi Arabia; tulbah@kfshrc.edu.sa

**Keywords:** PD-L1, formalin, FFPE, frozen, immunohistochemistry, breast cancer

## Abstract

Purpose: Response to anti-PD-L1/PD-1 immunotherapy correlates with PD-L1 expression in breast cancer. However, the prevalence of PD-L1 positive breast cancer is variable, which could be due to differences in the population/cohort of patients tested or the preservation/detection technology used. To investigate this variability, we examined the effect of two tissue preservation methods on PD-L1 immunohistochemical detection in breast cancer. Methods: We compared PD-L1 expression in patient-matched frozen (FR) and formalin-fixed paraffin-embedded (FFPE) tissues of breast cancer patients. PD-L1 expression was assessed using tumor proportion score (TPS, simply PD-L1 score), and case positivity was determined with PD-L1 score ≥5. Results: In FFPE tissues, PD-L1 was positive in 7–10% of tested patients, depending on the antibody used. In patient-matched FR tissues, the same antibodies showed positive PD-L1 expression in 20–30% of cases. The impact of the antibody tested on the rate of PD-L1 positivity (% of PDL1 positive cases) was minor, as evident in the near perfect concordance between PD-L1 score obtained using the different antibodies whether tested in FR or FFPE tissues. However, there was a systematic drop by an average of 13–20% in the PD-L1 score obtained in FFPE tissues compared to their patient-matched FR tissues. Conclusions: In the tested patient-matched cohort, there was consistently a higher PD-L1 score in FR than FFPE tissues, regardless of the antibody used, demonstrating a significant effect on PD-L1 detection due to the preservation method. These findings should inspire further work to improve the sensitivity of PD-L1 detection and possibly search for more sensitive antibodies in FFPE tissues.

## 1. Introduction

Programmed death-ligand 1 (PD-L1) is a T-cell inhibitory molecule that is expressed on antigen-presenting cells (APC), leading to the induction of T-cell anergy and/or apoptosis [1]. PD-L1 is aberrantly overexpressed in most malignancies (reviewed in [2]) to promote their immune escape, and therapies against PD-L1 showed unprecedented response rates in cancer patients (reviewed in [3]). Importantly, the status of PD-L1 expression correlates with a positive response to anti-PD-L1 therapy [4]. Therefore, it is necessary to accurately assess PD-L1 status in tumors that respond to anti-PD-L1 immunotherapy, including breast cancer [5].

We and others have previously demonstrated the expression of PD-L1 in breast cancer and its correlation with well-known poor prognostic factors [6,7]. Subsequently, other studies have shown similar findings using much larger sample cohorts [7,8,9,10,11,12,13]. However, there was considerable variability between studies in the prevalence of PD-L1 positive cases in breast cancer. It remains to be determined whether this is due to differences in the population/cohort of patients tested or the preservation/detection technology used.

We previously reported a positive PD-L1 expression status in 28% of breast cancer cases (without selecting a specific subtype, Supplementary Table S1) [6]. This percentage was higher than what later was reported by other investigators within a range of 2 to 22% of breast cancer patients [8,9,10,11,12,13]. One of the main methodological differences between our previous study and later reported studies was the method of tissue fixation/preservation used. Our previous study used fresh frozen (FR) tissues [6,7], while most of the subsequent studies used formalin-fixed paraffin-embedded (FFPE) tissues, the standard method for the preservation of tissues from clinical samples. In addition, we used the MIH1 antibody, which is not compatible with FFPE tissue.

Currently, several FDA-approved anti-PD-L1 antibodies in FFPE tissues are available. In this study, immunohistochemistry (IHC) was used to evaluate PD-L1 expression in a cohort of breast cancer patients. We used currently available antibodies to compare PD-L1 expression in matched FR and FFPE tissues of breast cancer patients. IHC demonstrated a higher PD-L1 expression score in FR tissues than FFPE tissues.

## 2. Materials and Methods

### 2.1. Patient Selection and Consenting

This study was conducted under the Helsinki Declaration, and it was approved by the Research Advisory Council (RAC# 2140-001) of King Faisal Specialist Hospital and Research Centre. Sections from archived paraffin-embedded breast cancer samples were obtained from the full cohort of 69 patients diagnosed with invasive ductal carcinoma of the breast. Sections from archived fresh FR breast cancer tissues were available from a subcohort composed of 30 patients (tissues of the other 39 patients were exhausted). All patients signed an informed consent form approved by KFSH&RC, as previously described [6,14].

### 2.2. Tissue Fixation and Embedding

FR tissues were collected, flash-frozen in liquid nitrogen while being embedded in optimal cutting temperature (OCT) compound, and stored at −80 °C as previously described [6,14].

FFPE tissues, which were fixed in 10% neutral buffered formalin (NBF) for 24–48 h, were collected and embedded in paraffin using the Tissue-Tek^®^ automated paraffin embedding system (Sakura, Torrance, CA, USA) and archived as a routine procedure for cancer tissue collected for clinical diagnosis.

### 2.3. Antibodies

Three different, previously characterized, and well-validated antibodies (E1L3N, SP263, and 28-8) were used in addition to the MIH1 antibody. The E1L3N clone (Cat# 13684, Cell Signaling Technology, Danvers, MA, United States) was chosen due to its higher reported sensitivity compared with other clones [15]. On the other hand, SP263 (Cat# 07494190001, Ventana, Oro Valley, AZ, United States) was chosen as the standard anti-PD-L1 antibody used by Ventana BenchMark autostainer, available in our pathology department. On the other hand, the 28-8 clone (Cat# ab205921, Abcam, Cambridge, United Kingdom) is a companion test used to detect PD-L1 for the therapeutic anti-PD-L1 Nivolumab^®^ (Bristol Meyers Squibb, BMS, New York, NY, USA). The MIH1 (Cat# 14-5983-82, eBioscience, San Diego, CA, USA) was the primary antibody used in our previous studies [6,7].

### 2.4. Immunohistochemistry (IHC)

#### 2.4.1. IHC on Sections of FR Tissues

IHC of FR tissues have previously been described in detail [6,14]. Briefly, tissue sections (5 µm) were fixed in acetone before incubation with MIH1 or 28-8 antibodies (15 and 10 min, respectively). For the E1L3N antibody, FR tissue sections were initially fixed in 50% methanol in PBS, followed by 4% formaldehyde for 6 min.

Tissue sections were incubated overnight with 1:70 (7 µg/mL), 1:200 (4 µg/mL), and 1:800 (1.3 µg/mL) diluted MIH1, E1L3N, and 28-8 antibodies, respectively. Ready-to-use Envision Flex (Agilent DAKO, Santa Clara, CA, USA) was used as a secondary antibody, and 3,3-diaminobenzidine (DAB, Agilent Dako) was used as a substrate.

#### 2.4.2. IHC in Sections of FFPE Tissues

IHC, using anti-PD-L1 (SP263) in FFPE tissues, was done using Ventana benchmark Ultra, a fully automated platform, and staining was completed as per their instructions using OptiView DAB IHC detection kit (Ventana). Briefly, sections were baked at 75 °C for 4 min, antigen retrieval was done using cell conditioning 1 (CC1, Ventana) solution for 64 min. Sections were incubated with the SP263 primary antibody for 28 min at 36 °C, followed by OptiView HQ Linker for 8 min and OptiView HQ Multimer for another 8 min. Counterstain was carried out using Hematoxylin II.

IHC, using E1L3N and 28-8 antibodies in FFPE tissues, was done manually according to the manufacturer’s instructions, as shown in Supplementary Table S2. Briefly, tissues were sectioned (5 microns), dewaxed, and rehydrated. Antigen retrieval was conducted using CC1 solution in the Decloaking Chamber pressure cooker (Biocare, Pacheco, CA, USA). The primary antibodies were incubated overnight in a humidified chamber at dilutions of 1:200 (4 µg/mL) and 1:500 (2 µg/mL) for E1L3N and 28-8 antibodies, respectively. Envision G/2 or Flex polymer (ready-to-use, Dako, Agilent) was used as a secondary antibody, and DAB was used as a substrate.

Initially, all antibodies and their respective recommended protocols were verified using sections from FFPE blocks of MDA-MB-231 and MCF-7, as positive and negative controls, respectively, for PD-L1. All validated antibodies gave a specific, intense membranous staining in MDA-MB-231 and were negative for MCF-7 (Supplementary Figure S1).

### 2.5. Pathology Scoring

An anatomical pathologist (AT) scored PD-L1 expression in FR and FFPE tissue sections using 5 to 10 increments to generate a tumor proportional score TPS, which is defined as a percentage of positive tumor cells in relation to total tumor cells in the examined field. PD-L1 positivity of breast cancer cases was determined using a unified cutoff of 5%, as reported previously [6], irrespective of the PD-L1 staining intensity. PD-L1 expression in tumor-associated macrophages, dendritic cells, and lymphocytes was ignored.

### 2.6. Statistical Analysis

Agreement of scores was determined using Lin’s concordance correlation coefficient (C; using actual PD-L1 scores), and the interpretation of concordance was done as reported previously [16]. Using data of MIH1 on FR sections as a comparator, sensitivity and specificity were calculated using the standard equations: sensitivity = TP/(TP + FN), while specificity = TN/(TN + FP) were TP = True positive, TN = True negative, FP = False positive, FN = False negative. The statistical analyses were performed using SAS 9.4 (Statistical Analysis System, SAS Institute Inc., Cary, NC, USA) or GraphPad Prism (La Jolla, CA, USA). A two-sided *p*-value of < 0.05 was considered statistically significant.

## 3. Results

### 3.1. Lower Prevalence of PD-L1 Positive Tumors in FFPE Compared to FR Tissues

Initially, we used the SP263 antibody (FDA-approved diagnostic antibody on a fully automated testing platform) as a reference for the detection of PD-L1 expression status in FFPE tissues of our previously reported cohort of 69 patients. PD-L1 expression results obtained with SP263 antibody in FFPE tissues were then compared with the previously reported results for MIH1 antibody in their patient-matched FR tissues. PD-L1 was positive in 13% of patients using SP263 antibody in FFPE tissues as compared to 28% obtained with MIH1 antibody in patient-matched FR tissues.

We have further tested the correlation of PD-L1 expression obtained with SP263 antibody in FFPE tissues with hormone receptor status of breast cancer, known prognostic markers to correlate with PD-L1 expression in breast cancer. We found that PD-L1 obtained with SP263 antibody in FFPE tissues correlated with estrogen and progesterone receptor-negative status in a similar fashion to what was previously reported with the MIH1 antibody in FR tissues (Table 1).

**Figure 1:**
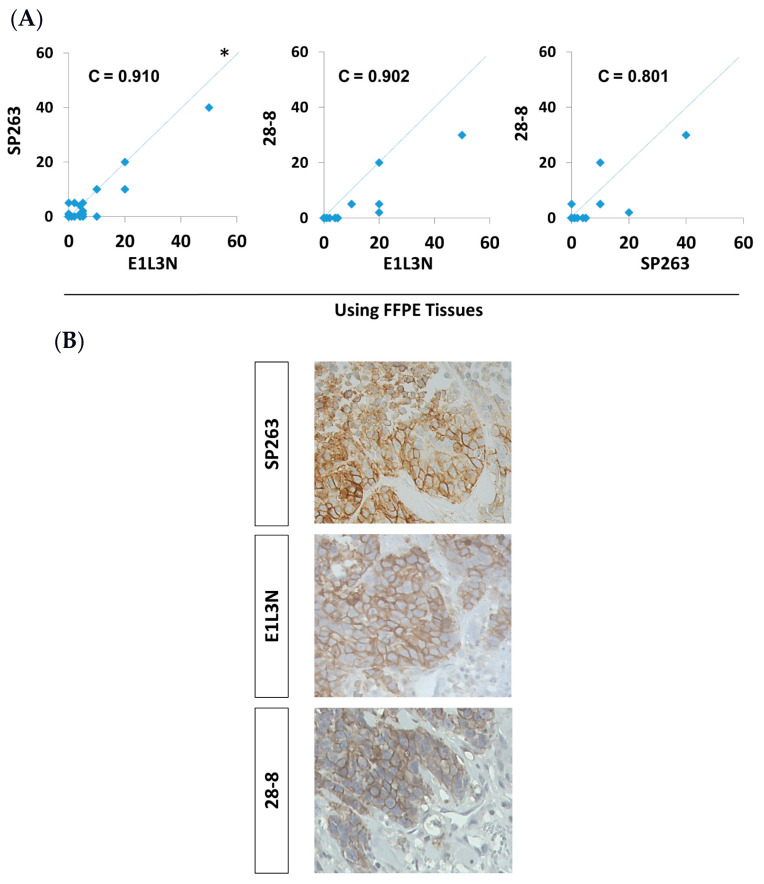
(**A**) Pairwise comparison of PD-L1 score between E1L3N, 28-8, and SP263 antibodies in FFPE tissues in a cohort of 69 patients. Comparison of PD-L1 tumor proportional score (TPS) between different antibodies in 69 patients. C = concordance correlation coefficient. * The dashed line represents the 45-degree hypothetical perfect-match line. (**B**) Representative images of immunohistochemical detection for PD-L1 (brown) using three different antibodies in sections of FFPE tissues and examined under a light microscope at ×400 magnification.

As the reduced PD-L1 expression rate in FFPE compared to FR tissues could have been due to the antibody used or the preservation method, we tested other PD-L1 antibodies that are compatible with FFPE tissue. There was a comparable positivity rate of 9 and 17% of patients for 28-8 and E1L3N antibodies, respectively. More specifically, there was a high concordance (concordance correlation coefficient, C = 0.8 to 0.91) between the actual PD-L1 scores obtained with the different antibodies tested in FFPE tissues (Figure 1A,B). Altogether, the prevalence of PD-L1 positive breast cancer cases using the three antibodies was around 13% using FFPE tissues, which is lower than previously reported with FR tissues.

In order to test the effect of tissue preservation on PD-L1 detection while maintaining the antibody used, we searched for PD-L1 antibodies that can work in both FR and FFPE tissues. Unfortunately, the previously used antibody (MIH1) in FR tissues was incompatible with FFPE tissues, and the SP263 antibody was incompatible with FR tissues, making them unsuitable for such a comparison. On the other hand, E1L3N and 28-8 antibodies, which are compatible with FFPE tissues, were found to be suitable for FR tissues as well, upon method optimization. Therefore, these two antibodies were used on a subcohort of 30 patients (limited by the availability of FR tissues from the original cohort of 69 patients) to assess the expression of PD-L1 in FR and FFPE tissues side-by-side.

### 3.2. PD-L1 Score and Prevalence of PD-L1 Positive Cases in FR Tissues Were Comparable within All Tested Antibodies

**Figure 2:**
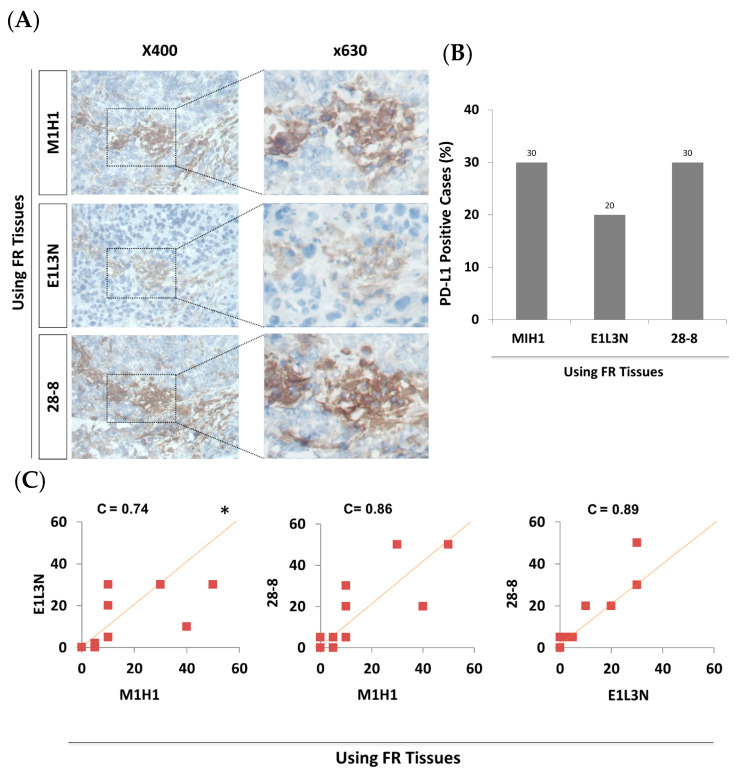
Comparable PD-L1 IHC detection in FR tissues of the subcohort (30 breast cancer patients) using MIH1, E1L3N, and 28-8 antibodies. (**A**) Representative images of immunohistochemical detection for PD-L1 (brown) using three different antibodies in sections of FR tissues and examined under a light microscope at ×400 and ×630 magnifications. (**B**) Bar graph showing the prevalence (rate) of PD-L1 positive breast cancer patients (%) in FR tissues using three different antibodies. (**C**) Pairwise comparison of the actual PD-L1 score (TPS) between the three antibodies in FR tissues. C = concordance correlation coefficient. * The dashed line represents the 45-degree hypothetical perfect-match line.

The three tested antibodies showed similar areas of staining in matched sequential sections of FR tissues (Figure 2A). Importantly, the expression of PD-L1 was mainly membranous/cytoplasmic, as reported previously. Prevalence of PD-L1 positive cases in FR tissues was 30% after retesting with MIH1 antibody in the subcohort of 30 patients (Figure 2B), which is comparable to the 28% previously reported with the full cohort of 69 patients [6]. Similarly, PD-L1 was positive in 20–30% of cases in FR tissues using E1L3N and 28-8 antibodies, respectively. Pairwise comparison of the actual PD-L1 score between the different antibodies tested in FR tissues showed a substantial to perfect concordance (C = 0.74–0.89) (Figure 2C). Altogether, all tested antibodies showed comparable reactivity to PD-L1 in FR tissues.

### 3.3. PD-L1 Score and Prevalence of PD-L1 Positive Cases in FFPE Tissues Were Comparable within All Tested Antibodies

**Figure 3:**
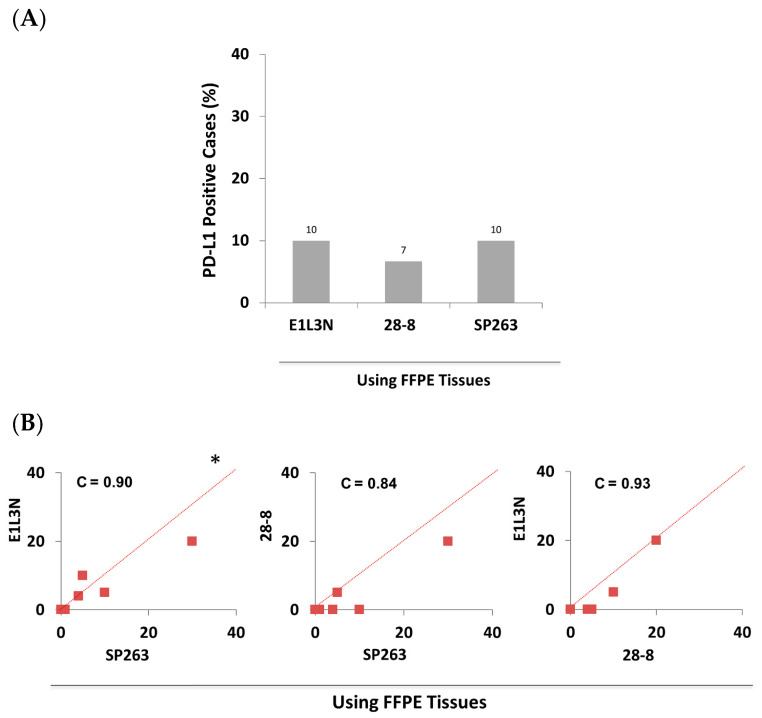
Comparable PD-L1 IHC detection in FFPE tissues of the subcohort (30 breast cancer patients) using SP263, E1L3N, and 28-8 antibodies. (**A**) Bar graph showing the prevalence (rate) of positive breast cancer patients (%) in FFPE tissues using three different antibodies. (**B**) Pairwise comparison of the actual PD-L1 score (TPS) between the three antibodies in FFPE tissues. C = concordance correlation coefficient. * The dashed line represents the 45-degree hypothetical perfect-match line.

In patient-matched-FFPE tissues, PD-L1 was positive in 7–10% cases of the subcohort of patients using 28-8 and E1L3N antibodies, respectively (Figure 3A), which was considerably below the patient positivity rate observed in FR tissues. Pairwise comparison of the actual PD-L1 scores between the different antibodies tested in FFPE tissues showed a perfect concordance (C = 0.84–0.93), irrespective of the antibody used (Figure 3B). The reduced prevalence of PD-L1 positivity observed in FFPE compared to FR tissues, regardless of the antibody used, strongly suggests that tissue preservation methods influenced the PD-L1 detection more than the antibody used.

### 3.4. The Drop in Prevalence of PD-L1 Positivity and Its Score in FFPE Tissues Compared to FR Tissues Is Consistent within All Tested Antibodies

**Figure 4:**
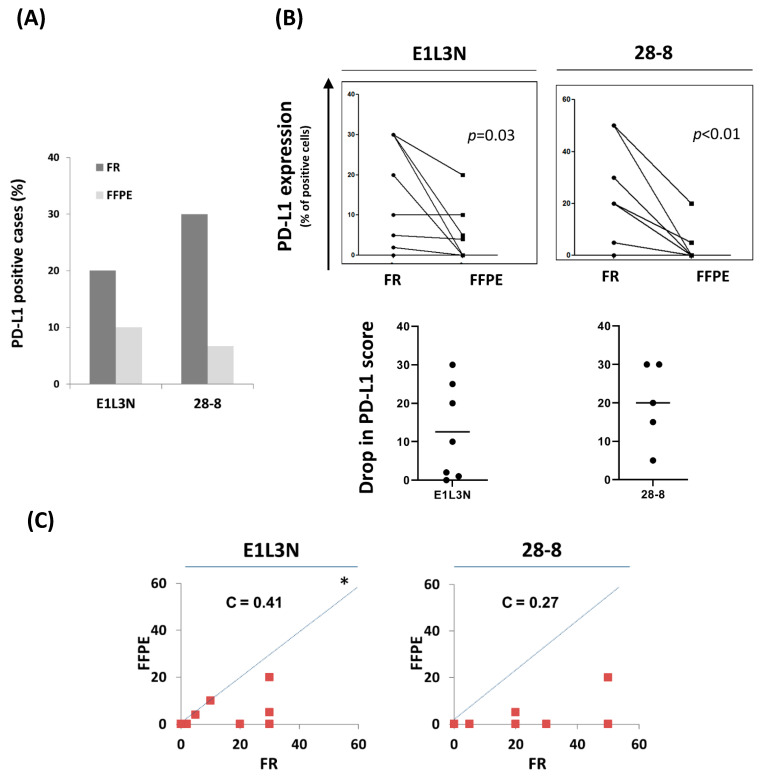
Higher PD-L1 IHC detection in FR compared to patient-matched FFPE tissues of the subcohort (30 breast cancer patients) using E1L3N and 28-8 antibodies. (**A**) Bar graph showing the prevalence (rate) of positive breast cancer patients (%) in FR and FFPE tissues using E1L3N and 28-8 antibodies. (**B**) Dot plots showing a pairwise comparison of actual PD-L1 score, TPS, between E1L3N and 28-8 antibodies in FR tissues (top). Dot plots for PD-L1 score drop in FFPE vs. FR tissues, the line in the middle represents the average (bottom). (**C**) Pairwise comparison of the PD-L1 score, TPS, between E1L3N and 28-8 antibodies in FR tissues. C = concordance correlation coefficient. * The dashed line represents the 45-degree hypothetical perfect-match line.

In order to examine the effect of tissue fixation/preservation type on the reactivity of each anti-PD-L1 antibody, we compared the rate of PD-L1 positivity between patient-matched FR and FFPE tissues using either E1L3N or 28-8 antibodies, separately. In the subcohort of 30 patients tested, the rate of PD-L1 positive cases was 2–3 times higher in FR compared to FFPE tissues (Figure 4A). Pairwise comparison of the actual PD-L1 scores in patient-matched FR and FFPE tissues showed a statistically significant drop in PD-L1 score. The drop in PD-L1 score was observed for both antibodies, with an average score decrease by 13 and 20% for E1L3N and 28-8 antibodies, respectively (Figure 4B). Concordance between PD-L1 scores in FR and FFPE tissues was fair, and most of the data points were below the 45-degree hypothetical perfect-match line, indicating higher reactivity to FR tissues compared to FFPE tissues (Figure 4C). Altogether, our data from patient-paired analysis demonstrated a lower rate of PD-L1 positivity in FFPE than FR tissues, irrespective of the tested antibody.

### 3.5. The Decrease in Sensitivity of Anti-PD-L1 Antibodies in FFPE Tissues Is Predominantly due to the Preservation Method

The above data demonstrated a lower reactivity of anti-PD-L1 antibodies and a lower PD-L1 score in FFPE as compared to FR tissues. In order to further evaluate the effect of tissue preservation, we calculated the sensitivity/specificity of E1L3N and 28-8 antibodies in FFPE tissues using the MIH1 antibody as an independent comparator. This approach was carried out to discriminate between differences due to antibodies and those related to the preservation method.

Our results showed a change in the sensitivity that could be attributed to a difference in the antibody used, which would contribute to the observed drop in PD-L1 detection in FFPE compared to FR tissues. For example, the E1L3N antibody showed a change in sensitivity by 33% (100 to 67%) compared with the MIH1 antibody (Table 2). There was a further decrease by 34% (67 to 33%) due to the use of FFPE tissues instead of FR tissues. Similarly, 28-8 antibody had an initial change in sensitivity of 11% (100 to 89%) compared with MIH1 antibody and a greater drop of 67% (89 to 22%) in FFPE instead of FR tissues. On the other hand, specificity was similar between the three antibodies and the relative specificity remained around 100% in FFPE tissues. Altogether, our data show a decrease in antibodies’ sensitivity by 34–67% in FFPE compared to FR tissues while the specificity was preserved, indicating that these differences were predominantly due to the preservation method.

## 4. Discussion

The temporal and spatial heterogeneity in PD-L1 detection has been well-described [17,18]. This heterogeneity has inspired several researchers to compare the performance of different anti-PD-L1 antibodies (reviewed by Eckstein et al. [19] and Buttner et al. [17]). However, methodological variability, such as the effect of the tissue preservation method, has not been elucidated. In this study, we evaluated the effects of two standard methods of tissue preservation on PD-L1 detection by IHC in one cohort of breast cancer patients. Using patient-matched tissues, we have demonstrated significantly higher PD-L1 scores in FR tissues compared to their matched FFPE tissues.

Although it is the standard of care for pathological diagnosis worldwide, FFPE tissues have a major limitation, which is the loss of antibody sensitivity to an antigenic epitope following formalin fixation, a well-known phenomenon [20,21]. However, antibodies can be generated against epitopes that are more stable with formalin fixation. Alternatively, the antigenicity can be retrieved with common antigen retrieval methods so the effect of formalin fixation, or other treatments that are part of paraffin embedding, including dehydration with ethanol and clearing with xylene, could be reversed or minimized [22]. While FR tissues are not practical to use in the clinic, it is important to know how much sensitivity is lost to antigens in FFPE compared to FR tissues and whether more sensitive antibodies should be developed. PD-L1 protein, in particular, is more sensitive to formalin fixation than other proteins as it contains only two small linear hydrophilic regions and, therefore, a limited number of antibody binding sites are available [23,24]. Indeed, previous studies have hinted at the decrease in antibody reactivity to PD-L1 in FFPE compared to FR sections [25,26]; however, these studies did not investigate this decrease further using the newly available anti-PD-L1 antibodies.

Several studies have shown the interchangeability of E1L3N, SP263, and 28-8 anti-PD-L1 antibodies in FFPE tissues [27,28], which is consistent with our results. Interestingly, Tsao et al. [27] have shown a similar signal intensity using the quantitative immunofluorescence technique. However, these studies were limited to FFPE tissues. We have shown that the PD-L1 score is consistently higher in FR tissues, suggesting there is room for improving the sensitivity of PD-L1 immunohistochemical detection. Indeed, there is evidence that the sensitivity of current IHC methods for detecting PD-L1 can be improved in FFPE by deglycosylation [29]. Notably, when the FDA approved the different anti-PD-L1 antibodies for their clinical use and according to Martinez-Morilla et al., “they required proof of quality, reproducibility, and sensitivity for prediction, but were less stringent on analytic sensitivity” [28]. Furthermore, available summaries of safety and effectiveness data (SSED) for the FDA-approved antibodies SP263 and 28-8 lack comparative studies for their performance in FR and FFPE tissues [30,31].

We and others have reported on the expression of PD-L1 in breast cancer patients [9,10,11]. However, the range of reported prevalence of PD-L1 positive breast cancer cases has been quite large, ranging from 1.7% to as high as 57%. The exact cause of this variability is not well-known. Several suspected factors for the variability in PD-L1 expression among various studies include the population studied, the methodology employed, and the antibody used. In this study, we have used patient-matched breast cancer samples to test the effect of tissue preservation methodology on measuring PD-L1 expression level. We have demonstrated using patient-matched tissues that PD-L1 detection is more sensitive in FR than FFPE tissue. Our findings of PD-L1 sensitivity loss in FFPE tissues, in addition to other factors such as the antibody used, might account for most of the variability in the previously reported prevalence of PD-L1 positive breast cancer patients.

A limitation in our study is the lack of a true reference for PD-L1 expression in FR tissues. In addition, none of the patients in this study received anti-PD-L1 immunotherapy; thus, we could not assess the correlation between patient response and PD-L1 status using FR vs. FFPE tissues. However, the consistently higher scores of PD-L1 in FR compared to FFPE tissues, irrespective of the antibody tested, support the loss of PD-L1 sensitivity in FFPE tissues. Future trials can be arranged to address these issues and confirm whether PD-L1 positive tumors in FR tissues only would correlate with the patient response. Further work could correlate PD-L1 mRNA with protein expression in FR and FFPE using techniques like RNAscope [32]. However, it is important to keep in mind that the expression of PD-L1 mRNA does not necessarily translate into protein due to a well-known post-transcriptional regulation of PD-L1 [33].

The population of breast cancer patients might also play a role in the prevalence of PD-L1 positivity of breast cancer patients. Locally advanced breast cancer (LABC), a more aggressive form of breast cancer, tends to have a higher prevalence in Saudi Arabia [34]. Obesity is another factor prevalent in Saudi society [35] and tends to be linked to an increased risk of postmenopausal breast cancer [36]. Whether these factors affect PD-L1 expression is not well-known. Future studies are needed to simultaneously compare this Middle Eastern population side-by-side with other populations using the same methodology.

In summary, we used IHC to compare different validated antibodies and fixation methodologies on PD-L1 detection using a patient-matched breast cancer cohort. The findings reported in this study demonstrated a higher sensitivity of PD-L1 in FR as compared to FFPE tissues. This study recommends developing more sensitive anti-PD-L1 antibodies for FFPE tissues and stresses the need to compare FFPE with FR tissues during antibody validation.

## Figures and Tables

**Table 1:** Correlation of PD-L1 expression with estrogen receptor and progesterone receptor status as detected by SP263 antibody in FFPE tissues and MIH1 antibodies in FR tissues.

	PD-L1 (SP263)			PD-L1 (MIH1)		
	−	+	*p	−	+	*p
Estrogen Receptor Status						
Positive	44 (94)	3 (6)		37 (79)	10 (21)	
Negative	16 (73)	6 (27)	**0.025**	12 (55)	10 (45)	**0.039**
Progesterone Receptor Status						
Positive	33 (97)	1 (3)		28 (82)	6 (18)	
Negative	27 (77)	8 (23)	**0.028**	21 (67)	14 (33)	**0.041**

The estrogen and progesterone receptor status obtained from patients’ files as described previously in detail [6,7]. *p values in bold represent significant data.

**Table 2:** Specificity and sensitivity of validated anti-PD-L1 antibodies in FR and FFPE tissues of 30 breast cancer patients.

	E1L3N			28-8		
	FR	FFPE	↓Drop	FR	FFPE	↓Drop
♣ R. Sensitivity	67	33	34	89	22	67
R. Specificity	100	100	0	95	100	0

♣ Sensitivity and specificity are relative to MIH1 reactivity in FR sections.

## Data Availability

The datasets generated during and/or analyzed during the current study are included in this published article (and its supplementary information files), otherwise available from the corresponding author on reasonable request.

## Supplementary Materials

The following are available online at https://www.mdpi.com/article/10.3390/antib10030024/s1, Figure S1: Performance of the three anti-PD-L1 antibodies in FFPE sections, Table S1: Distribution of the molecular subtypes of breast cancer cases, Table S2: Conditions used for manual Immunohistochemical detection of PD-L1 on FFPE tissues.

## Abbreviations

PD-L1	programmed death ligand-1
FR	Frozen
FFPE	Formalin-Fixed Paraffin-Embedded
IHC	Immunohistochemistry
C	Lin’s concordance correlation coefficient
