# Supplementary material for: Higher PD-L1 Immunohistochemical Detection Signal in Frozen Compared to Matched Paraffin-Embedded Formalin-Fixed Tissues
Source: Antibodies (Basel). 2021 Jun 22;10(3):24. doi: 10.3390/antib10030024 (PMC8293136; doi:10.3390/antib10030024)
Supplement: Supplementary file 1 [file antibodies-10-00024-s001.zip › antibodies-1160023-supplementary.pdf]

Supplementary Table 1: Distribution of the molecular subtypes of breast cancer cases

Type	Luminal A	Luminal B	Her 2	TN
Number of Patients	25 (36)*	22 (32)	10 (14)	12 (17)

* number in brackets represents the percentage of cases

Supplementary Table 2: Conditions used for manual Immunohistochemical detection of PD-L1 on FFPE tissues

Antibody	Antigen Retrieval		Antibody diluent	Antibody dilution	Incubation	Secondary antibody
	Solution	Conditions				
E1L3N	CC1	121°C /6 min	CS (#8114)	1:200	OVN	Envision G/2
28-8		110 C/ 10 min	0.1% BSA	1:500		Envision Flex

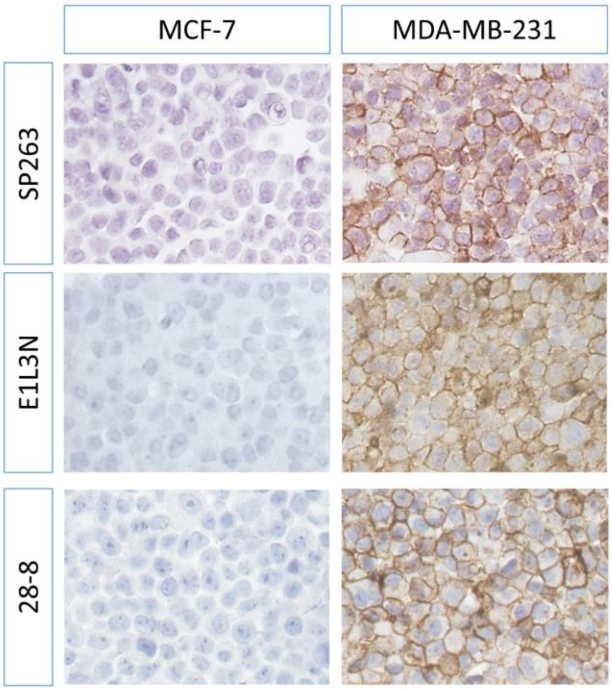

Figure 1 Performance of the three anti-PD-L1 antibodies in FFPE sections. Representative images of immunohistochemical detection for PD-L1 (brown) using three different antibodies in sections of FFPE sections of positive (MDA-MB-231) and negative (MCF7) controls examined under a light microscope at x400 magnification.

Supplementary figure 1
